# The role of sex as a biological variable in neurocritical care: Fundamentals of cerebrovascular physiology with clinical implications

**DOI:** 10.1177/0271678X261447779

**Published:** 2026-05-13

**Authors:** Lauren E Maier, Jatinder S Minhas, Cornelia Hoedemaekers, Philip N Ainslie

**Affiliations:** 1 Center for Heart, Lung, and Vascular Health, School of Health and Exercise Sciences, University of British Columbia—Okanagan, Kelowna, BC, Canada; 2 Cerebral Haemodynamics in Ageing and Stroke Medicine (CHiASM) Research Group, Department of Cardiovascular Sciences, University of Leicester, Leicester, UK; 3 NIHR Leicester Biomedical Research Centre, British Heart Foundation Centre of Research Excellence, Glenfield Hospital, Leicester, UK; 4 Department of Intensive Care, Radboud University Medical Centre, Nijmegen, The Netherlands

**Keywords:** Cerebral blood flow, cerebral ischemia, cerebrovascular disease, hormones, sex

## Abstract

Ischemic stroke, intracerebral hemorrhage (ICH), aneurysmal subarachnoid hemorrhage (aSAH), global hypoxic ischemic brain injury following cardiac arrest (HIBI), and traumatic brain injury (TBI) present clinical pathologies that share brain ischemia as a primary or secondary consequence of their respective pathophysiology. Recently, sex—as a biological variable—has received attention for its clear implications in differences in injury pathophysiology, diagnosis, and management. Due to historical, social, and gender biases, females have been excluded from physiology-based research and clinical trials, resulting in limited insight into female physiology and poor translation of current diagnostics and therapies to females. The role of sex-specific hormones has been implicated in a host of sex differences following cerebrovascular injury and the ensuing clinical outcomes. Therefore, this review aims to provide a contemporary overview on the role of biological sex and sex hormones in the underlying physiology, mechanisms, and outcomes of selected cerebrovascular injuries. First, we identify sex-based differences in clinical outcomes in ischemic stroke, ICH, aSAH, HIBI, and TBI. Second, we discuss the impact of biological sex and sex hormones on the fundamentals of cerebrovascular physiology. Finally, related mechanisms of sex-based differences in injury pathophysiology are explored to identify potential therapeutic avenues for future research.

## Background

Cerebral injuries underpinning neurocritical care encompass entities that are often grounded in the pathophysiologic cascade of brain parenchymal lesions, ischemia, inflammation, and their downstream sequelae. Specifically, ischemic stroke, intracerebral hemorrhage (ICH), aneurysmal subarachnoid hemorrhage (aSAH), global hypoxic–ischemic brain injury following cardiac arrest (HIBI), and traumatic brain injury (TBI) present clinical pathologies that may present with brain ischemia as either a primary or secondary consequence of their respective pathophysiology. Thus, the understanding and regulation of clinical factors that govern cerebral oxygen delivery are paramount for clinicians.

In recent years, research investigating sex—as a biological variable—has shown clear implications for differences in the pathophysiology, diagnosis, and management of ischemic injury.^1^ Sex refers to biological differences between males and females, whereas gender encompasses social and cultural factors related to a range of gender identities from man to woman, in a particular historical and cultural context. Historically, due to social and gender biases, there has been disproportionate physiology-based research targeting males versus females,^1^ leading to limited insights into female clinical physiology and inadequate translation of currently available diagnostics and therapies to females.^2^ Although sex is a fundamental aspect of human physiology, this essential biological variable is rarely considered in the design of basic physiological studies, in translating findings from basic science to clinical research, or in developing personalized medical strategies.^3^ In particular, the impact of hormonal differences and various organ-specific effects of estrogen, progesterone, and other related sex hormones (e.g. testosterone, luteinizing hormone) is of paramount importance in discriminating female from male physiology.

Within this context, current epidemiologic studies demonstrate that female sex is a key biological variable in the associations between cerebrovascular injury and clinical outcomes. For example, females have a greater incidence of stroke and aSAH and experience worse outcomes, including reduced quality of life and increased disability.^2,4^ Risk of ischemic stroke significantly increases in females following menopause,^5^ implicating sex-specific hormones in disease risk and outcomes. Given these links to adverse outcomes in females and a role of sex as a biological factor in acute neurologic disease severity, we sought to provide a narrative review on the underlying physiology, mechanisms, and clinical outcomes. While this review focuses on sex as a biological variable, gender-based differences are equally important considerations for clinical outcomes. Specifically, the aims of our narrative review are to: (1) describe sex-based differences in clinical outcomes in neurocritical care disease entities; (2) discuss the impact of biological sex and sex hormones on the fundamentals of cerebrovascular physiology; and (3) explore the potential physiologic mechanisms of biological sex on acute cerebrovascular injury pathophysiology to identify therapeutic avenues of research.

## Sex in neurocritical care disease entities and cerebrovascular injury

### Ischemic stroke

Stroke, and the downstream ischemic sequelae, account for 10% of total deaths worldwide.^6^ Until recently, differences in clinical outcomes between sexes were not widely reported; however, it is increasingly recognized that following ischemic stroke, females often exhibit worse long-term neurologic outcomes, higher mortality, and reduced quality of life.^2,7,8^ Despite these clear sex-specific differences, the underlying mechanisms are poorly understood. Epidemiologically, females presenting with ischemic stroke are older^2,7^ and less frequently receive thrombolytic intervention compared to males, despite having similar eligibility.^9^ In contrast, recent evidence suggests significantly higher incidence of ischemic stroke in young (⩽35 years) females compared to males (incidence rate ratio: 1.44 (95% CI: 1.18–1.76); reviewed in Leppert et al.^10^). Pre-clinical evidence has suggested female sex hormones may be protective following cerebral ischemia through a myriad of influences (e.g. reducing ischemic damage through smaller infarct volumes and reduced inflammation), and appear to be important for reducing ischemic stroke risk and improving outcomes (reviewed in Sohrabji et al.^5^). Chronic changes in these hormones, such as menopause or hormonal contraceptive use, significantly increases stroke risk.^5,8^ However, an increased risk of stroke in premenopausal females suggests there are other important female-specific risk factors (i.e. maternal strokes, hormonal contraceptive use, polycystic ovary syndrome) to consider.^10^ In addition, females are more susceptible to traditional risk factors, including diabetes, hypertension, and atrial fibrillation.^3,8^ Clearly, differences between males and females in ischemic stroke are inextricably linked to a myriad of factors, both specific and generalized across the sexes.

### Intracerebral hemorrhage

ICH is the second most common stroke subtype, characterized by its high mortality rate (>50%).^11^ Males have a greater incidence of ICH, and at a younger age,^11^ in part due to earlier onset of cerebral amyloid angiopathy and a greater likelihood of ICH following cerebral amyloid angiopathy in males.^12^ Other risk factors of ICH (i.e. hypertension, smoking, and alcohol abuse) are more common in males.^11^ Despite some protection against ICH incidence, females experience more severe ICH and higher mortality.^11^ There is an important relationship between aging and ICH risk, across sexes and populations.^13^ However, this may be more pertinent in females. For example, Foschi et al.^11^ reported that age predicted 30-day and 1-year fatality in females but not in males. With aging in females, and concomitantly, menopause, ICH risk increases.^14^ Sex hormones in menopause appear to be related to ICH incidence and outcomes,^11,14^ but the specific influence remains to be understood.

### Aneurysmal subarachnoid hemorrhage

Although aSAH represents a minority of all strokes (~5%), its high risk of mortality, reduced quality of life, functional impairments, and disproportionate burden on females render it crucial to improve treatments and outcomes.^4^ Females are more susceptible to cerebral aneurysms and, therefore, experience a higher prevalence of aSAH.^4,15,16^ In addition to more aSAHs, females may also experience higher rates of delayed cerebral ischemia, a serious complication following aSAH (reviewed in Fuentes et al.^4^). Despite this, aSAH outcome severity does not appear to be related to sex.^4,16^ In contrast with ischemic stroke, traditional risk factors for aSAH including hypertension, smoking, and coronary artery disease were associated with a similar increase in aSAH risk between males and females, while diabetes mellitus was suggested to reduce aSAH risk in females only.^17^ Age is an important contributor to aSAH occurrence in females. Females are older at aSAH presentation compared to males and are even more likely to experience aSAH following menopause.^16,17^ Sex hormones may contribute to these relationships through changes with menopause,^16–18^ but their exact role requires further research.

### Hypoxic ischemic brain injury

HIBI occurs in comatose patients after cardiac arrest and involves a cascade of negative processes resulting in secondary brain injury and cell death from reduced blood flow and oxygen deprivation (reviewed in Sekhon et al.^19^). HIBI classification is the primary determinant of outcomes in post-cardiac arrest patients,^19^ and this may be exacerbated in females, who experience higher mortality and unfavorable complications with secondary ischemia.^20^ Females are reported to have worse neurological outcomes and chances of survival following cardiac arrest, present with more non-shockable rhythms, and receive fewer resuscitative medications compared to males.^21^ Further, an interaction between age and sex on cardiac arrest outcomes^22^ suggests a role for sex hormones. In support, animal models have shown estrogen is protective against global neuronal ischemia,^23^ but there is a gap in translation into human clinical understandings of sex differences in HIBI pathophysiology.

### Traumatic brain injury

TBI presents an alternate etiology to the aforementioned neurocritical disease entities that results in ischemic injury. Epidemiological data suggests males are 40% more likely than females to experience a TBI.^24^ Conversely, females experience higher mortality and worse outcomes, including greater severity, more post-concussive symptoms, and worse motor function, particularly following mild to moderate TBI.^24^ Worse neurological and functional outcomes in females has been associated with greater cerebral edema and differences in inflammatory cytokines.^25^ Unlike nontraumatic brain injuries, TBI incidence decreases with age. Albeit more prevalent in males, worsened TBI outcomes in females appear to be influenced by puberty, menstrual cycle phase, and menopause.^24^ Further, TBI may disrupt the normal menstrual cycle; many females experience amenorrhea (i.e. absence of menstruation) following injury, and injury severity is predictive of the length of amenorrhea.^26^ Although amenorrhea is common following a variety of stressful episodes (e.g. mental trauma, high levels of exercise, and significant weight loss), this finding still highlights a key link between TBI and female hormones. Clearly, it is imperative to understand how sex and sex-specific hormones interact with TBI pathophysiology.

### Other sex-specific cerebrovascular syndromes

Although rare and accounting for <1% of all strokes,^27^ cerebral venous and sinus thrombosis (CVST) affects females at a ratio of 3:1 compared to males.^28^ There are key gender-specific risk factors influencing CVST prevalence, such as use of oral contraceptives, pregnancy, postpartum, and hormone replacement therapy.^29^ Despite higher prevalence, females may have better prognosis following CVST compared to males, which may in part be due to a younger age at incidence.^30^ Another syndrome, reversible cerebral vasoconstriction, is the sudden constriction of segments of the intracranial arteries coupled with a severe, recurrent headache and is significantly more common in females.^31^ Reversible cerebral vasoconstriction syndrome is a common cause of stroke, particularly in younger adults. Research suggests it may be triggered by pregnancy and puerperium factors, including eclampsia and preeclampsia.^31^

## Sex and cerebrovascular physiology

**Figure 1.**
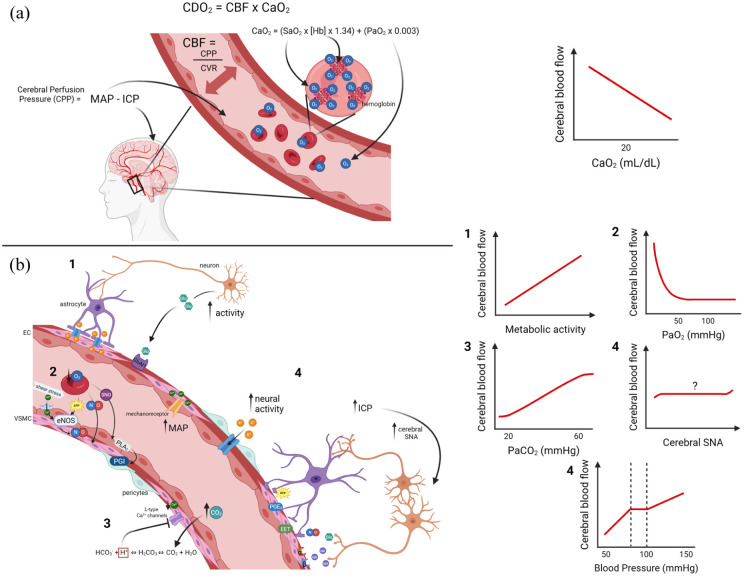
(a) Determinants of cerebral oxygen delivery. CDO_2_ is directly proportional to CBF and CaO_2_. CaO_2_ is determined by the SaO_2_ in the blood, the concentration of Hb, and the maximum amount of oxygen that can bind to one Hb (1.34 mL), added to the amount of oxygen, that is dissolved in plasma (PaO_2_ × 0.003). CBF is directly proportional to CPP and inversely proportional to CVR. CPP is determined by MAP and ICP. CVR is a measure of the vascular tone and is regulated by multiple local and central mechanisms, many of which are detailed in panel (b) and (b) mechanisms of CBF regulation. (1) *Neurovascular coupling:* increases in neuronal activity are transmitted through astrocytes to the vascular wall via increases in [Ca^2+^]. BK channels on astrocyte endfeet enable movement of K^+^ and Ca^2+^ with VSMCs, triggering relaxation through inward rectifying potassium channels. Neurons also mediate vasodilation directly through release of Glu, which binds to GluN1 receptors on VSMCs. (2) *Hypoxia-mediated vasodilation:* reduction in the PaO_2_ triggers production of NO in red blood cells, endothelial cells, and neurons, which diffuses into VSMCs or pericytes. eNOS is triggered through increases in [Ca^2+^] via shear stress-activated mechanoreceptors. SNO are transported from red blood cells to the endothelium, which can release NO to trigger vasodilation. Red blood cells also release ATP in hypoxia, which activates endothelial purinergic P2_Y2_ receptors, increasing intracellular Ca^2+^ release and stimulating eNOS. Endothelial membrane phospholipids activate PLA_2_, triggering a cascade of events resulting in PGI production. (3) *Cerebrovascular reactivity to CO_2_:* increases in PaCO_2_ mediate changes in extracellular pH. CO_2_ molecules move across the blood–brain barrier, where they are converted via carbonic anhydrase into carbonic acid, then bicarbonate and H^+^. Reductions in pH from accumulation of H^+^ reduce L-type Ca^2+^ channel activity, thus reducing VSMC [Ca^2+^] and enabling relaxation. (4) *Autonomic control and cerebral autoregulation:* increases in ICP increase cerebral sympathetic nerve activity, resulting in NA release from the axon terminal, activating adrenergic receptors. α1 and postjunctional α2 receptors increase VSMC [Ca^2+^] and vessel tone. NA can also bind to β receptors. β1 receptors trigger vasoconstriction, whereas β2 receptor activation results in vasodilation. Neurons and astrocytes also intrinsically mediate vessel tone. Neurons release Glu, which activates a cascade of events within the astrocyte producing PGE_2_ or EETs, two vasodilators. Similarly, ATP increases [Ca^2+^] in the astrocyte, triggering release of the vasodilator K^+^. NO release triggers neuronal Glu release, which causes relaxation of VSMCs. Increased [K^+^] in the endothelial cells from neural activity hyperpolarizes the cells, which is propagated to VSMCs through gap junctions. Finally, increases in intravascular blood pressure are sensed by mechanically sensitive voltage-gated Ca^2+^ channels. An influx of Ca^2+^ depolarizes the VSMCs and causes vasoconstriction. Created with Biorender.com. CaO_2_: arterial oxygen content; CBF: cerebral blood flow; CDO_2_: cerebral oxygen delivery; CPP; cerebral perfusion pressure; CVR: cerebrovascular resistance; EETs: expoxyeicosatrienoic acid; eNOS: endothelial NO synthase; Glu: glutamate; Hb: hemoglobin; ICP: intracranial pressure; MAP: mean arterial pressure; NA: noradrenaline; NO: nitric oxide; PaO_2_: partial pressure of oxygen; PGE_2_: prostaglandin E_2_; PGI: prostaglandin I; PLA_2_: phospholipase-A2; SaO_2_: saturation of oxygen; SNO: S-nitrosothiol; VSMCs: vascular smooth muscle cells.

**Figure 2.**
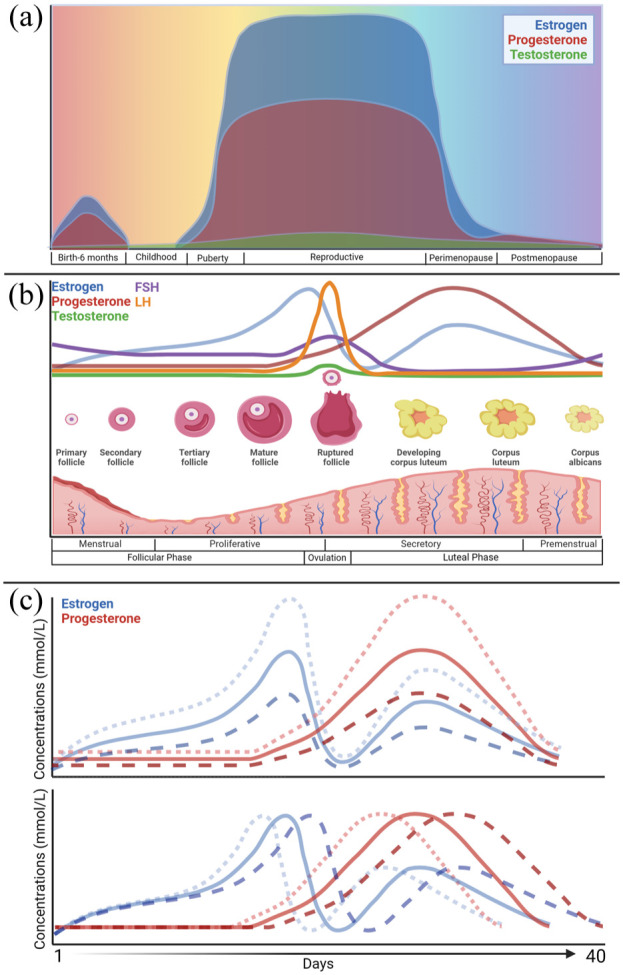
(a) Estrogen, progesterone, and testosterone levels throughout a female’s lifespan, (b) estrogen, progesterone, testosterone, FSH, and LH levels across one menstrual cycle. The cycle begins with the start of menstruation, with the shedding of the endometrial lining. During the proliferative phase, an ovarian follicle ripens in the ovaries into an ovum and the endometrium becomes thicker and more vascular. The ovum is released during ovulation and transported through the fallopian tubes to the uterus, becoming the corpus luteum. In the secretory phase, the endometrial glands become swollen with secretions. If the ovum is not fertilized, the endometrium breaks down and menstruation occurs, and (c) normal interindividual variation in estrogen and progesterone concentrations and length of menstrual cycle. Menstrual cycle length can vary considerably within a normal range of 21–40 days, as illustrated by D’Souza et al.^150^ Created with Biorender.com. FSH: follicle-stimulating hormone; LH: luteinizing hormone.

The brain is dependent upon constant oxygen and nutrient delivery to sustain homeostatic functions. Cerebral oxygen delivery is directly proportional to cerebral blood flow (CBF) and arterial oxygen content (CaO_2_). CBF is directly related to cerebral perfusion pressure and inversely proportional to cerebrovascular resistance. The regulation of CBF is complex, heterogeneously distributed across brain regions, and governed by multiple, clinically relevant pathways (reviewed by Willie et al.^32^ and Claassen et al.^33^). As illustrated in Figure 1, there are four/five overlapping factors that act to regulate CBF including neurovascular coupling, arterial blood gases, autoregulation, and the autonomic nervous system. These key regulators of CBF may be influenced or modified by sex and sex-specific hormones. Throughout the menstrual cycle, endogenous sex hormones including estrogen and progesterone are continuously fluctuating (Figure 2); however, the specific hormone levels are highly variable both within and between individuals and cycles.^34^ Thus, it is difficult to isolate the role of these hormones on CBF regulation and, as a result, there are conflicting findings. A brief overview of each of these CBF regulatory processes, including the fundamental anatomy, is provided with particular focus on the potential sex differences and effects of sex hormones in these responses.

### Fundamental anatomy

Structurally, the brain is comprised of distinct anatomical foci encompassing gray and white matter. Specifically, regions composed of gray matter include the cerebral cortex, thalami, and basal ganglia, which exhibit higher resting local perfusion compared with sub-cortical white matter. These regions of gray matter require more energy and, therefore, higher blood flow for synapses and action potentials, compared to white matter, where myelination significantly improves energetics of their synapses and action potentials.^35^

Anatomically, females may exhibit a greater gray-white matter ratio compared to males,^36^ although this is not a universal finding.^37^ Additionally, females exhibit greater cerebral cortical thickness, but regional differences are complex and area-specific.^38^ For example, the frontal lobe receives significant perfusion in both sexes, but is even greater in females.^39^ Gray matter volume may differ across the menstrual cycle; a study using magnetic resonance imaging (MRI) found a peak in total gray matter volume during ovulation.^40^ De Bondt et al.^41^ identified a strong, negative correlation between estradiol and gray matter volume of the anterior cingulate cortex in naturally cycling females that was not present in contraceptive users. White matter microstructure also differs between sexes; females have higher tract complexity and white matter dispersion, while males have greater directional sensitivity.^38^ Subcortical volumes are likely influenced by sex, specifically throughout the aging process, but are also segment-specific and highly variable.^38^ Males have a larger volume of the thalamus, as well as the globus pallidus and putamen of the basal ganglia, but other structures (i.e. caudate nucleus and nucleus accumbens) are similar between sexes.^38^

### Resting CBF and metabolism

The physiologic role of sex on CBF regulation is notable with its intersection on these anatomical structures and regulatory CBF mechanisms. For example, females present with smaller total brain volume compared to males.^36,42^ However, evidence suggests males may experience aging-related atrophy to a greater degree than females;^43^ thus, sex differences in brain volume may narrow with aging. Such anatomical differences, if they do occur, may contribute to the consistent finding of higher resting CBF in females. This observation that CBF is higher in females when compared to males is predicated upon both regional and global assessment methods including arterial spin labeling MRI,^42^ 4-dimensional flow MRI,^39^ positron emission tomography (PET),^44^ Xenon gas-based neuroimaging,^45^ and transcranial doppler ultrasonography.^46^ Critically, CBF may be up to 40% higher in females, when important confounders (i.e. age, comorbidities, smoking, medications, and menstrual cycle phase) are controlled for.^42^ Whether resting CBF differs across the menstrual cycle is unclear; some evidence suggests no influence of cycle phase on CBF assessed via transcranial doppler ultrasonography.^47^ In contrast, another study utilizing transcranial doppler ultrasonography found an increase of ~12% in middle cerebral artery velocity in the late compared to early follicular phase,^48^ and a third study assessing CBF via MRI^49^ reported that CBF was 10% higher in the follicular versus luteal phase. Cote et al.^50^ identified regional, opposing relationships between estradiol and progesterone with CBF in menstruating females, which may contribute to the variability in menstrual cycle phase results. Thus, changes in CBF throughout the menstrual cycle and the interplay with hormone concentrations requires further investigation.

In addition to structural differences, it is widely known that CaO_2_ is slightly lower in females owing to the impact of anemia, hormones, and other related factors (e.g. menses).^42^ To compensate for a lower CaO_2_, in order to maintain a stable cerebral oxygen delivery, resting CBF is elevated. Indeed, reduced CaO_2_ stemming from anemia is related to increased CBF responses,^51^ which is regulated by neuronal and glial cell metabolism at the level of the neurovascular unit.^32^ Specifically, Muer et al.^42^ found males had significantly elevated CaO_2_, likely due to higher hemoglobin, as hemoglobin was inversely associated with resting CBF (*r* = −0.68; *p* < 0.001). Importantly, young males and females appear to demonstrate similar cerebral metabolic rate of oxygen consumption (CMRO_2_),^44^ albeit others suggest higher CMRO_2_ in females.^52^ Thus, females may exhibit higher resting CBF to match a similar CMRO_2_ compared to age-matched males.^42^ However, these results should be interpreted with caution as estimated using MRI^42,52^ and PET scans,^44^ which rely heavily on biophysical models that require assumptions about vascular geometry, physiology (e.g. hematocrit, CaO_2_), and coupling between flow, volume, and metabolism, all which can vary across subjects and conditions. Recently, a large database of direct cross-brain sampling reported no difference between sexes in oxidative metabolism (assessed with oxygen-to-glucose and oxygen-to-carbohydrate ratios).^53^ In summary, as noted above, the higher resting CBF in females is likely due to a combination of structural differences and fluctuating hormone concentrations, resulting in lower hemoglobin concentrations and CaO_2_. Following ischemic stroke, younger females also have higher intracranial velocity (i.e. middle cerebral artery velocity) which is similarly related to hemoglobin levels,^54^ suggesting the robustness of these differences even throughout disease.

### Neurovascular coupling

The matching of cerebral metabolism with brain perfusion is referred to as neurovascular coupling. Activation of distinct anatomical foci in the brain results in a regional increase in CBF to meet its metabolic demands. In contrast to resting CBF, sex may not influence neurovascular coupling to the same extent.^55^ However, Koep et al.^56^ identified a negative relationship between peak posterior cerebral artery velocity responses with age in females (*p* = 0.005), but not males (*p* = 0.82), suggesting an important role of sex in neurovascular coupling throughout the lifespan. In this study, menopausal status was independently related to peak posterior cerebral artery velocity and cerebrovascular resistance index.^56^ Very limited research has investigated a role of the menstrual cycle, but Li et al.^57^ indicated potential regional differences between early and late follicular phases in neurovascular function. In females who experience repeated TBI, neurovascular coupling may be impaired;^58^ therefore, it is important to explore the interplay between sex and ischemic brain injury on neurovascular coupling to better inform therapies targeting brain metabolism and perfusion.

### Arterial blood gases

Changes in arterial blood gases, specifically the partial pressures of carbon dioxide (PaCO_2_) and oxygen (PaO_2_), trigger changes in CBF and vascular tone. Even small (1–2 mmHg) changes in PaCO_2_ alter extracellular pH by enabling movement of CO_2_ across the blood–brain barrier.^32^ Altered pH in the cerebral spinal fluid stimulates concomitant changes in the vasomotor tone of the cerebral vessels, thus, altering CBF. There seems to be no consensus on the effect of sex on cerebrovascular reactivity to CO_2_. For example, evidence exists suggesting higher cerebrovascular reactivity to elevations in PaCO_2_ in females,^59^ similar between males and females,^47^ or higher in males.^60^ These studies also explored how fluctuating hormone levels may influence CO_2_ reactivity; Skinner et al.^59^ identified blunted responses to hypocapnia during the early follicular compared to the ovulatory phase, but this difference had no impact on reported sex differences when compared with males in this study. In contrast, Favre and Serrador^47^ found no influence of menstrual cycle on cerebrovascular reactivity to CO_2_. Clinically, a recent meta-analysis found impaired CO_2_ reactivity in patients following stroke and TBI, but whether sex influences this relationship has not been investigated.^61^

In contrast to changes in PaCO_2_, the cerebrovasculature is relatively insensitive to arterial hypoxemia. For example, it is generally not until PaO_2_ is reduced below ∼50 mmHg that vascular tone is reduced and vasodilation occurs (referred to as hypoxia-mediated vasodilation). As reviewed in depth elsewhere,^62^ this hypoxia-mediated vasodilation elevates CBF and acts to maintain cerebral oxygen delivery. Hypoxia-mediated vasodilation may be greater in females due to lower hemoglobin concentrations,^63^ although this is not universal.^46^ One study investigating how menstrual cycle phase may influence the response to hypoxia found similar responses across the early and late follicular phases, despite greater basal middle cerebral artery velocity in the late follicular phase.^48^ How—and if—the cerebrovascular responses to hypoxia clinically may differ between males and females remains underexplored.

### Autonomic control and cerebral autoregulation

Cerebral perfusion pressure is largely determined by the difference of mean arterial pressure (MAP) with intracranial pressure; thus, changes in MAP directly influence cerebral perfusion pressure, altering CBF. Cerebral autoregulation (CA) is a clinically important mechanism triggering changes in cerebrovascular resistance in response to changes in system blood pressure to maintain CBF and cerebral oxygen delivery demands.^32^ This encompasses both acute, transient changes (dynamic CA) and slower, sustained pressure changes (static CA). The final and related, albeit subtle, regulator of both CBF and CA is the autonomic nervous system (reviewed by Koep et al.^64^). Autonomic control of the cerebrovasculature is nuanced and dependent on various factors such as receptor type and density, neurotransmitter concentration, and vessel location to maintain adequate perfusion.^64^ Sympathetic activation results in the release of neurotransmitters to act upon the vasculature through adrenergic receptors but can have conflicting actions of dilation or constriction.^64^

A recent meta-analysis exploring dynamic CA found greater 0.05 Hz middle cerebral artery coherence and smaller gain values in healthy young males compared to females during squat-stand maneuvers.^65^ Further, regardless of menstrual cycle phase, females demonstrate a smaller change in middle cerebral artery velocity in exposure to changes in blood pressure compared to males, suggesting a preserved state of dynamic autoregulation.^47^ Indeed, CA may be relatively unaffected by the menstrual cycle,^66^ although oral contraceptive use may alter autoregulation.^67^ The physiological significance and implications of these findings is unclear. Clinically, however, CA dysfunction following aSAH may be related to functional recovery and outcomes,^68^ but an interaction between CA and sex in aSAH has not been investigated. As reviewed by Mankoo et al.,^69^ autonomic control of the cerebrovasculature, particularly CA, is impaired following stroke. However, very little is known clinically about how the autonomic system influences CBF. To date, no research in health or disease has explored the role of sex in the autonomic control of CBF and whether sex contributes to cerebral autonomic control is also unknown.

### Menopause and aging considerations

During menopause, females experience significant changes in chronic levels of sex hormones (Figure 2). Intriguingly, postmenopausal females with a typical onset of menopause have lower resting CBF and cerebrovascular conductance compared to their premenopausal counterparts.^70^ These findings extend to clinical conditions; Mazzucco et al.^54^ found lower CBF in older, late postmenopausal females following minor ischemic stroke. In addition, CBF regulatory responses may also differ with menopause. Limited evidence suggests cerebrovascular reactivity to CO_2_ may be reduced in healthy peri- and postmenopausal females.^71^ The age at menopause onset may be an important consideration as females who undergo menopause at a younger age have reduced CO_2_ reactivity.^72^ In contrast, pre- versus postmenopausal status may not influence CA in otherwise healthy females.^70^ Interestingly, hormone replacement therapy does not appear to restore CBF to premenopausal values.^59^ As such, the precise mechanisms by which menopausal status impacts CBF regulation may be attributable to additional variables beyond purely sex hormones.

### Aging

It is important to consider the interplay between sex and aging and differentiate from age-related changes in hormones (i.e. menopause). With aging, the adult brain undergoes gradual reductions in mass, volume, and synaptic density.^37,73^ Albeit females showing smaller absolute brain volume across the adult lifespan,^38^ evidence suggests males may experience aging-related atrophy to a greater degree.^43^ Thus, sex differences in brain volume may narrow with aging. CBF also decreases with aging and in proportion to gray matter volume,^74^ although menopause may accelerate CBF reductions in females.^75^ Compared to age-matched males, postmenopausal females appear to have similar resting CBF,^76^ suggesting a significantly greater decline in females with aging, which is inextricably tied to menopause. Age-related changes in cerebrovascular resistance, endothelial function, and the blood–brain barrier contribute to increased risk of ischemic injury^77^; if females experience these changes in cerebrovascular regulation to a greater degree, it may contribute to the significant elevation in disease risk in older females. Similar to CBF, regional CMRO_2_ may decrease with aging, albeit it may be to a lesser extent than CBF changes, resulting in increased oxygen extraction in these regions of the brain (e.g. motor cortex, primary sensory, and temporal cortices).^78^ How these factors of brain metabolism interact with aging and sex is unclear.

Aging also influences the blood flow response to changes in metabolism. NVC responses decrease with aging, but this appears only in females.^56^ Further, reduced peak NVC responses in older females was only evident in later postmenopausal females, and menopausal status remained a significant factor when aging was controlled for. Thus, there appears to be both distinct effects and an interplay of aging and hormonal status on NVC. Similarly, many studies support a reduction in CO_2_ reactivity with aging (reviewed in Hoiland et al.^79^), and aging may have a greater effect in females,^80^ albeit challenged by others.^81^ Galvin et al.^82^ identified alterations in resting middle cerebral artery velocity and reactivity to hypocapnia with aging that were related to ischemic stroke risk, suggesting an important relationship with CO_2_ reactivity, aging, and disease risk. As identified by Carr et al.,^62^ there is a scarcity of data on how hypoxia-mediated vasodilation is influenced by aging in both sexes. In contrast to other factors that regulate CBF, cerebral autoregulation may be preserved with healthy aging; despite increases in systemic blood pressure, the cerebrovasculature appears to modulate resulting changes in intracranial pressure relatively well.^33^ It is unclear how sex or sex-related hormones might influence this response.

## Proposed mechanisms underpinning sex differences in cerebral ischemia

In all the reviewed disease entities and cerebrovascular injuries, outcomes in females are related to aging, particularly surrounding the period of menopause (Table 1).^2,8,11,13,17,22,24^ This is no coincidence; estrogen, progesterone, and testosterone contribute to physiological differences that underpin these functional outcomes. Further, evidence in both naturally fluctuating (e.g. pregnancy, puberty) and externally manipulated hormones (e.g. contraceptives, hormone replacement therapy) highlight the importance of these hormones in female disease outcomes. Importantly, sex-specific hormones have both organizational (irreversible influences) and activational (effects are dependent of presence or absence of the hormone) functions^83^ that underpin responses to both global and localized cerebral ischemia (Table 2).

**Table 1.** The effect of biological sex on prevalence and outcomes of neurocritical care disease entities.

Disease	Differences in disease entities	Proposed mechanisms
Ischemic stroke	Higher prevalence in older females^151^	Age, age at menopause, estrogen, testosterone, contraceptive use, increased susceptibility to diabetes, hypertension, atrial fibrillation
	Potential higher incidence in younger females ⩽35^10^	Female-specific risk factors: maternal strokes, hormonal contraceptive use, polycystic ovary syndrome
	Often worse mortality reported in females^2,151,152^	Age, menopausal status, estrogen, progesterone, testosterone, differences in treatment
	Females often have greater severity strokes^152^	Pre-stroke disability, age, estrogen, progesterone, testosterone, differences in treatment
	Reported that females have lower health-related quality of life, greater disability, more physical impairments and activity limitations^2,7,151^	Pre-stroke disability, age, estrogen, progesterone, testosterone, differences in treatment
	Higher poststroke depression in females^7^	Social isolation, pre-stroke depression, age, estrogen
Intracerebral hemorrhage	Greater incidence in males^11,153^	Estrogen, progesterone, testosterone, behavioral/lifestyle factors (e.g. alcohol abuse, smoking), genetics, increased risk of cerebral amyloid angiopathy
	Reports of greater severity in females^154^	Pre-stroke disability, age, menopausal status, estrogen, progesterone, testosterone, differences in treatment, pregnancy
	Reports of higher mortality rate in females^11^	Pre-stroke disability, age, menopausal status, estrogen, progesterone, testosterone
	Higher incidence of deep vein thrombosis following ICH in females^153^	Menopausal status, estrogen, pregnancy
	Worse neurological outcomes in females related to greater severity^153,155^	Age, pre-stroke disability, menopausal status, estrogen, progesterone, differences in treatment
	Females are more likely to have lobar ICH, while males are more likely to have deep ICH^11,155^	Prevalence of hypertension, age, estrogen, genetics
Aneurysmal subarachnoid hemorrhage	Greater prevalence in females^4,17^	Age, menopausal status, estrogen, progesterone, testosterone, contraceptive use, number of pregnancies
	Females have a greater incidence of delayed cerebral ischemia and hydrocephalus^4,156^	Age, menopausal status, estrogen
	Reports of worse functional outcomes in females^156^	Age, menopausal status, catecholamine concentrations, estrogen
	Greater incidence of multiple aneurysms in females related to higher prevalence of aSAH^4^	Age, menopausal status, estrogen
	Females have more internal carotid or posterior communicating artery aneurysms, while males have more anterior cerebral artery aneurysms^4^	Artery diameter/anatomy, estrogen
Hypoxic ischemic brain injury	Greater incidence of cardiac arrest and HIBI in males^20^	Age, menopausal status, estrogen, progesterone, testosterone
	Reports of lower survival following cardiac arrest and HIBI in females^20^	Age, shockable rhythm, differences in treatment, estrogen, progesterone, testosterone
	Worse neurological outcomes in females related to lower survival^21^	Age, differences in treatment, ventricular size, estrogen, progesterone
	Preclinical evidence suggesting reduced severity of ischemia–reperfusion injury in young females^23,157^	Estrogen, progesterone
Traumatic brain injury	Significantly greater incidence in males (up to 40%)^24^	Age, behavioral/lifestyle factors
	Incidence reports suggest females are more likely to experience sports-related concussions and TBI from assault and intimate partner violence^158^	Age, behavioral/lifestyle factors, biomechanical differences, contraceptive use
	Higher mortality rate in females following mild-moderate TBI^24^	Estrogen, progesterone, intracranial pressure, cerebral edema and inflammatory cytokines
	Reports of worse neurological outcomes in females^24,159–161^	Age, menopausal status, estrogen, progesterone, inflammatory pathways, contraceptive use
	Lower quality of life following TBI in females related to poorer neurological outcome and greater severity^160,161^	Age, estrogen, progesterone, contraceptive use

**Table 2.** Summary of the effects of sex hormones on cerebral ischemia.

Hormone	Impact on cerebral ischemia	Considerations
Estradiol	• Inhibits inflammatory response to ischemia^83,85,88,89,162,163^	• Evidence mainly from pre-clinical animal models^83,85–89,100,162–166^
	• Attenuates tumor necrosis factor^83^	
	• Upregulates brain-derived neurotrophic factor^167^	• In humans, timing of estradiol therapy may be crucial to its effects^97^
	• Increases transcription of “pro-survival” (Bcl) gene^85^	
	• Reduces transcription of “pro-apoptotic” (Bax) gene^85^	
	• Reduces blood–brain barrier disruption^85,88,89,162,163^	
	• Improves ATP synthesis in brain mitochondria^85,89,162,163^	
	• Reduces reactive oxygen species release^85^	
	• Enhances vasodilation in cerebral arteries through increases endothelial nitric oxide synthase activity and prostacyclin levels^89,162,163^	
	• Reduces neuronal cell death from global ischemia^87,100^	
	• Inhibits autophagy and promotes Nrf2–ARE transcription pathway^164^	
	• Promotes neuronal survival through PI3K/Akt/GSK3 pathway^85,89,162,163,166^	
	• Reduces GFAP and reactive gliosis following ischemia^166^	
Progesterone	• Reduces ischemic injury size/infarct volume^101–104,168^	• Conflicting evidence suggests only beneficial in male rats^103^
	• Decreases neurological deficits^101,102,104^	
	• Reduces blood–brain barrier disruption^102^	• Benefit of progesterone in hormone replacement therapy is unclear^105^
	• Attenuates cerebral edema^102^	
	• Inhibits pro-inflammatory cytokine expression, including IL-1β and TGFβ2^102^	
	• Enhances brain mitochondrial function^102,156^	
	• Attenuates reactive oxygen species production^168^	
	• Protects from neuronal damage in the central nervous system^102,169^	
	• Improves brain-derived neurotrophic factor expression^169^	
Testosterone/DHT	• Impairs blood–brain barrier function^106^	• Conflicting evidence as hindering or helpful in ischemic response^106,107,109,110,170–174^
	• Increases neuronal cell death^106^	
	• Augments cerebral inflammation^106^	
	• Exacerbates ischemic injury^106,109,174^	• Positive effects may be age and dose-dependent^109^
	• Increases reactive astrocytes and microglia^170^	
	• May increase brain-derived neurotrophic factor^107,172^	• Differences may depend on applying testosterone as a treatment following injury vs circulating levels^106,109,113^
	• Protective against oxidative stress and improves mitochondrial function^107^	
	• Reduces apoptotic cell death^110,172^	• Differences may depend on conversion of testosterone to DHT or estradiol^109,111,112^
	• Improves mitochondrial function^110^	
	• Reduces ischemic infarct volume in older males^109^	
	• Promotes neurogenesis^172^	
	• Improves neurological outcomes^172,173^	

### Estrogen

There are three types of estrogen produced in the body (estrone, estradiol, and estriol). Estradiol (E2) is the most common and potent form in premenopausal females, whereas estrone (E1) is the main form postmenopause.^84^ Estrogens are primarily produced in the ovaries, but are also made in the liver, adrenal glands, mammary tissue, neurons, and adipose tissue. Production is triggered by release of gonadotropin-releasing hormone from the hypothalamus, causing release of luteinizing and follicle-stimulating hormones from the pituitary gland, in turn stimulating estrogen production. In premenopausal females, estradiol fluctuates with progesterone and other hormones throughout the menstrual cycle (Figure 2). Estradiol acts on the brain through estrogen receptors (ERα, Erβ, and G-protein estrogen receptor).^85^ ERα is the primary receptor for neuroprotection in cerebral ischemia. Estradiol replacement in ovariectomized in vivo rat models of middle cerebral artery occlusion showed a loss of neuroprotection with ERα deletion, but not ERβ.^86^ However, ERβ plays a crucial role in ischemia–reperfusion injury in ERβ knockout mice models.^86^ Nonselective ER antagonist both exacerbates cerebral ischemic injury in female rodents and blocks the protective effects of exogenous estradiol, highlighting the importance of these receptors in neuroprotection.^87^ Activation of these receptors located on neurons, astrocytes, microglia, and endothelial cells within the CNS triggers a range of functions in animal models including inhibition of the inflammatory response to ischemia, upregulation of brain-derived neurotrophic factor, and increased transcription of the “pro-survival” (B-cell leukemias and lymphomas (BCL)) and reduction of the “pro-apoptotic” (BCL2 associated X) genes.^85^ In humans, estradiol in premenopausal females may attenuate tumor necrosis factor, an important pro-inflammatory cytokine involved in many neurodegenerative diseases and elevated following stroke.^83^ Following preclinical middle cerebral artery ischemia–reperfusion, estradiol reduces blood–brain barrier disruption more than 50% and 30% in the cerebral cortex and subcortex, respectively.^88^ In ischemic rat brain mitochondria, estradiol is associated with improved ATP synthesis and reduced reactive oxygen species release.^85^ Further, estradiol increases vasodilation in cerebral rat arteries in vivo by promoting endothelial nitric oxide synthase activity and prostacyclin levels.^89^ Similarly to non-traumatic models of cerebral ischemia, estradiol treatment in rat TBI models reduces edema, inflammation, and intracranial pressure, enhances blood flow via vasodilation, and improves the integrity of the blood–brain barrier and vasculature.^90^ In humans, estradiol inhibits a key iron hormone hepcidin, contributing to anemia in menstruating females.^91^ The World Health Organization reported 30% of females aged 15–49 years experience anemia, likely owing to chronic iron depletion partly attributable to menses.^92^ Indeed, females consistently report lower hemoglobin levels compared to males. In ischemic stroke, lower hemoglobin levels in females are associated with greater neurological deficits, independent of the effect of sex.^93^ Thus, estradiol may actually contribute to worsened outcomes from ischemic stroke through indirect pathways. In contrast to estradiol, higher levels of estrone (postmenopausal estrogen), are related to poor neurological outcomes following global ischemia from cardiac arrest.^94^ Thus, not all estrogens have equal influence on the cerebrovasculature, and a better understanding of their differing influences is needed.

Overwhelming evidence in preclinical work supports a benefit of estradiol in neuroprotection and cerebral ischemic injury outcomes in all above disease entities. However, attempts to apply this knowledge to humans, specifically postmenopausal females, has shown mixed results. Consistently, premenopausal females show more favorable outcomes to various cerebral ischemic injuries compared to their postmenopausal and age-matched male counterparts.^8,14,17,22,72,85,89^ Yet, hormone replacement therapy with exogenous estradiol does not seem to improve outcomes following ischemic stroke, ICH, or aSAH in postmenopausal females,^95,96^ and some even suggest increased risk with estradiol supplementation.^97^ Other studies do support hormone therapy in improving neurological outcomes following cerebral aneurysm for postmenopausal females.^18^ One potential explanation for this large variability is the “Timing Hypothesis,” which states that the potential benefits and harms of hormone replacement therapy are related to the time of initiation following menopause.^97,98^ Indeed, research investigating early estradiol treatment in postmenopausal females has shown reduced risk of stroke,^99^ cardiac events,^97^ and neuronal cell death from global ischemia.^100^ This is likely linked to changes in estrogen receptors (ERα and ERβ) with menopause; as estrogen acts mainly through these receptors, their decreasing presence throughout menopause results in little to no effect of supplemental estrogen in years following.^98^ It is crucial to understand this window for estradiol supplementation and the potential wide range of benefits for neurological function, outcomes, and incidence of cerebral ischemic injuries.

### Progesterone

Progesterone is largely produced by the adrenal cortex and the gonads. Similar to estrogen, progesterone production is stimulated through a pathway starting with the release of gonadotropin-releasing hormone. Progesterone fluctuates throughout the menstrual cycle (Figure 2), markedly dropping off with menopause. Thus, it is intuitive progesterone may play a role in risk and outcomes following ischemic injury. Indeed, evidence suggests progesterone is neuroprotective acutely following stroke; progesterone receptor removal increased injury size and neurological deficits in mice.^101^ However, these benefits were more pronounced in male mice, suggesting progesterone may have a greater protective role in the male brain. Progesterone has pleiotropic effects following ischemia; evidence in rodents suggests reduced blood–brain barrier disruption, cerebral edema, pro-inflammatory cytokine expression, and improved brain mitochondria function.^102^ In contrast, progesterone lacks efficacy in ovariectomized female rodents, and thus may not be a crucial hormone for protection from ischemic insult in females.^103^ Conversely, Gibson et al.^104^ found progesterone supplementation to aging female rats was neuroprotective. In contrast to preclinical support for the efficacy of progesterone, a meta-analysis of progesterone treatment clinical trials in humans following TBI did not find improved neurological outcomes or mortality rates.^105^ Nevertheless, progesterone is often overlooked in understanding physiological sex differences in disease entities, but it may play a role in neuroprotection in both male and female brains. Moreover, many hormone replacement therapies are a combination of estrogen and progesterone; thus, both the independent role of progesterone and its interaction with estrogen need to be elucidated.

### Testosterone

Testosterone is produced mainly in the gonads, stimulated by the hypothalamic-pituitary-gonadal pathway, increasing in males with puberty and decreasing throughout aging. Testosterone can be converted to dihydrotestosterone (DHT) via the enzyme 5-α reductase and has more potent androgen effects. DHT may be related to worse ischemic injury, contributing to poorer outcomes in young males compared to age-matched females. In opposition of estrogen and progesterone, evidence in male rats suggests testosterone impairs blood–brain barrier function, increases neuronal cell death, and augments cerebral inflammation.^106^ In contrast, others suggest testosterone supplementation following stroke or cerebral ischemia improves rat recovery and outcomes.^107^ Fanaei et al.^107^ reported increases in brain-derived neurotrophic factor and protective effects against oxidative stress following preclinical brain ischemia. Many studies in humans have observed a decrease in testosterone following ischemic stroke, which could suggest (1) lower testosterone levels are related to stroke development or (2) the acute stress response to stroke triggers a decrease in testosterone.^108^ In elderly males following ischemic stroke, lower levels of testosterone were related to greater cerebral infarct size and loss of neural function.^108^ The role of testosterone may vary with age; in younger male mice, testosterone exacerbated ischemic injury, but reduced it in middle-aged mice.^109^ Following TBI, exogenous testosterone reduced cell death and improved mitochondrial function in mice.^110^ Further, the ratio of estradiol to testosterone may be important for ischemic stroke, as a higher ratio was associated with greater risk of acute ischemic stroke and poor functional outcomes in patients.^111^ Some animal models suggest that testosterone aromatization to estradiol is neuroprotective against traumatic and ischemic brain injury,^112^ while others report no role of cerebral aromatase, but rather androgen receptor signaling, in ischemic protection.^109^ Importantly, a prominent phase 4 clinical trial in older age males given testosterone or placebo gels was terminated early due to severe increase of cardiovascular-related adverse events.^113^ It was concluded that there may be a crucial difference in chronic testosterone levels as a risk factor and utilizing testosterone as a treatment. Therefore, there is a gap in translating the above-mentioned preclinical studies into clinically relevant studies in humans; as such, the role of testosterone in sex differences in ischemic injury remain unclear.

### HIF and erythropoiesis

There is some evidence for a role of sex hormones, particularly estradiol, in the hypoxic–ischemic pathway through hypoxia-inducible factor (HIF). HIF is a heterodimeric transcription factor, with an oxygen-sensitive α-subunit central to the cellular hypoxic response.^114^ There are three α-subunits: HIF-1α, HIF-2α, and HIF-3α. Together, HIF-1α and HIF-2α facilitate oxygen delivery and cellular adaptation to hypoxia and ischemia. During cerebral ischemic injury, HIF-1α is a key factor in the injury response; its activation regulates angiogenesis, glucose metabolism, and cell death.^115^ HIF-1α works by upregulating vascular endothelial growth factor (VEGF) and erythropoietin.^115^ Estradiol treatment in immature rats also induces expression of VEGF through recruitment of ERα and HIF-1α.^116^ Zheng et al.^117^ found estradiol treatment following preclinical ischemic stroke increased HIF-1α and VEGF levels, reducing brain injury and improving functional outcomes. Similarly, estradiol treatment of cerebral artery occlusion in rats resulted in enhanced HIF-1α that corresponded with increases in hemoglobin-α and -β protein levels in the hippocampus.^118^

**Figure 3.**
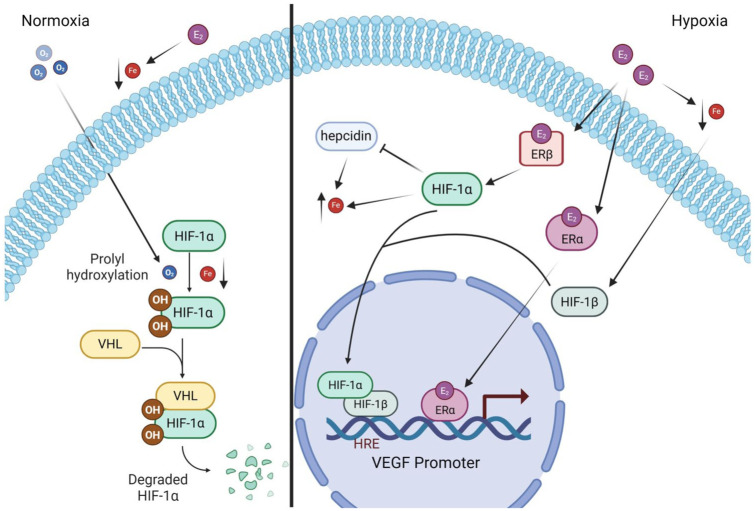
Proposed mechanisms of HIF, estrogen, and iron in a normoxic and hypoxic ischemic cell. In normoxia, HIF-1α is hydroxylated, leading to degradation. In hypoxia, hydroxylation is blocked; instead HIF-1α is phosphorylated and binds with HIF-1β to attack the hypoxia response element on the target gene, in this case the VEGF promoter. Estradiol further upregulates this pathway. First, estradiol binds to ERβ, which upregulates HIF-1α. In addition to increasing transcription, HIF-1α also downregulates hepcidin, which results in increased iron concentrations. HIF-1α also increases iron directly by increasing expression of iron transporters. Estradiol also binds to ERα, which may be able to directly influence transcription. Finally, extracellular estradiol reduces circulating iron concentrations. Low iron upregulates HIF-1β, increasing binding with HIF-1α and thus transcription. Created with Biorender.com.

HIF-2α is upregulated in anemia to stimulate erythropoietin.^119^ As estradiol plays a key role in regulating iron levels and contributing to anemia,^91^ there may be an important relationship between estradiol levels, anemia, and HIF in females that contributes to improved outcomes to cerebral ischemia in menstruating females (Figure 3). Qu et al.^120^ observed upregulation of iron transporters in microglia by estradiol through activation of HIF-1α. However, whether this relationship influences the response to cerebral ischemia and downstream sequelae has never been investigated. Lastly, the time course of cerebral ischemia may alter the effects of HIF. Following long-term hypoxia and ischemia via middle cerebral artery occlusion in rats, evidence suggests HIF-1α increases neuronal cell death and inflammation.^121^ There may be a critical window for the beneficial effects of HIF in cerebrovascular injury. A potential role of HIF and any interaction with sex-specific hormones in ischemic injury has never been investigated in humans.

## Influence on treatment

Clear sex differences exist in both the access to—and success with—various therapies for ischemic stroke^9^ and ICH.^122^ Females consistently demonstrate more non-traditional stroke signs and symptoms, which may contribute to slower and less frequent treatment.^9^ Mechanical clot retrieval via endovascular approaches and administration of thrombolytic drugs and tissue plasminogen activator have substantially advanced ischemic stroke treatment in the past three decades. However, females less frequently receive thrombolytic drugs compared to males, despite similar eligibility.^9^ This may be partly explained by greater stroke severity and older age at stroke onset in females; however, these are not absolute contraindications for intravenous thrombolytic intervention. Indeed, when given thrombolytic drugs, females report better outcomes compared to males.^9^ Evidence from preclinical models suggests the presence of estrogen in females improves these outcomes by reducing blood–brain barrier damage and preventing hemorrhagic transformation following thrombolytic intervention.^123^ Since 2015, females are just as likely to receive endovascular treatment as males; interestingly, however, females report worse functional outcomes but better mortality rates following treatment.^124^ Similarly, some evidence suggests females are less likely to receive various treatments for ICH, such as decompressive craniectomy and external ventricular drains.^122^ It remains likely that such differences in neurological outcome in survivors may also be related in decision making regarding end-of-life care and advance care planning.

In addition to receiving less treatment, the current therapeutic strategies for ischemic stroke, ICH, aSAH, and cardiac arrest are routed in evidence from clinical trials largely in males and may not translate fully to females. For example, there is some conflicting evidence in females supporting the use of aspirin. Research from nearly five decades ago suggests aspirin is ineffective as an antithrombotic agent in females,^125^ yet it is still routinely used as both primary and secondary treatments. Crucially, when considered without sex aggregated data, aspirin significantly decreases death and disability following ischemic stroke.^126^ As a strategy for stroke management, female sex has previously been associated with a poor response to the antiplatelet effects of aspirin.^127^ However, aspirin in secondary prevention has also been shown to reduce the risk of stroke in females by 17%, driven by a 24% reduction in ischemic stroke,^128^ suggesting it is a crucial therapy in females. Indeed, others have found aspirin to be effective in females as a secondary prevention for recurrent vascular events, including atherosclerotic cardiovascular disease^129^ and serious vascular and coronary events.^130^ A meta-analysis of aspirin use for secondary prevention of vascular events found a significant reduction in all vascular events that was similar between sexes.^130^ In contrast, in females without coronary heart disease, aspirin does not appear to reduce incidence of myocardial infarction or cardiovascular disease events,^128^ compared to a reduction in myocardial infarction incidence of 42% in males.^131^ Spranger et al.^125^ suggests testosterone may mediate platelet aggregation and the inhibition via aspirin; no difference in effectiveness of aspirin was observed pre- and postmenopause, indicating female sex hormones are not responsible. Compared to males, females may have higher clot strength and lower platelet inhibition response to mono-antiplatelet therapy (e.g. aspirin).^132^ There are key sex differences in pharmacokinetics that influence how many drugs, including aspirin, are metabolized (reviewed in Franconi et al.^133^). For example, CYP3A4, a key cytochrome P450 enzyme critical for prescription drug metabolism, is expressed at a higher level in females.^134^ Sex differences in CYP3A4 have a direct influence on the metabolism of direct oral anticoagulants (i.e. rivaroxaban and apixaban) used for ischemic stroke prevention in individuals with atrial fibrillation.^135^ Recent work from Foschi et al.^136^ in dual antiplatelet therapy found a lower frequency of 90-day new ischemic stroke or other vascular events following previous mild-to-moderate ischemic stroke or high risk transient ischemic attack in females compared to males. Importantly, aspirin is still an important therapy for secondary prevention of vascular disease events in females, but more research is needed to understand potential differences between sexes in platelet behavior and drug pharmacokinetics.

Vitamin K antagonists, such as warfarin, are key medications in the treatment of thromboembolic conditions, to which responses may differ by sex. For example, following warfarin treatment in patients with atrial fibrillation, females often experience a poorer International Normalized Ratio^137^ and spend less time in the therapeutic range.^138^ Preclinical models suggest females have both greater basal vitamin K-dependent clotting activity and longer half-lives for clotting factors II and X.^139^ Further, evidence from animal models indicates estradiol may inhibit the anticoagulant effects of warfarin.^140^ Changes in hormone concentrations may have crucial effects on coagulation; use of oral contraceptives is widely known to increase risk of thromboembolic events^141^ and the unique events of pregnancy, childbirth, and the postpartum period have significant impact on coagulation factors in females.^142^ These data emphasize the importance of individualized platelet function assessment and testing of the response to antiplatelet therapy, especially in females.

An important treatment that has significantly improved outcomes following aSAH is endovascular coiling. However, compared to males, females who underwent endovascular coiling were more likely to develop delayed cerebral ischemia and experienced additional sex-specific risk factors.^143^ Although speculative, Zhang et al.^143^ suggests estrogen may exhibit a heightened vasodilatory effect, leading to greater changes in cerebral perfusion. Albeit scarce, these data suggest sex hormones alter treatment pathways. It is crucial females and sex hormones are considered in future clinical trials and therapeutic strategy developments.

### Hormone therapy

In 2002, the results from the Women’s Health Initiative trial reported that hormone replacement therapy with conjugated equine estrogens and progestin increased risk of coronary heart disease, breast cancer, stroke, and pulmonary embolism,^144^ resulting in premature stopping of the trial. Coupled with the findings of more coronary heart disease events in the first year of the Heart and Estrogen/Progestin Replacement Study,^145^ hormone replacement therapy use rapidly decreased and doubt and uncertainty became widespread. In relation to cerebrovascular function, the Women’s Health Initiative Memory Study found increased incidence of dementia, mild cognitive impairment, and increased lesion volumes with hormone therapy.^146,147^ Critically, a large body of research since these trials has found the benefits and harms of hormone replacement therapy are related to the time of initiation following menopause onset.^97,98,148^ For instance, there may be reduced risk of cardiac death with early initiation of hormone therapy, albeit the profound effects of the original trial findings have significantly limited research in hormone replacement therapy. Significantly more research is needed to understand how exogenous hormone supplementation influences cerebrovascular physiology and disease risk and whether there is a potential future therapeutic use of hormone therapy.

In a different context, more recent research from gender-affirming hormone therapy has investigated how estrogen supplementation influences cerebrovascular outcomes in transgender adults.^149^ Transgender females taking estrogen may have an increased risk of ischemic stroke and cerebrovascular disease compared to cisgender populations.^149^ However, gender-affirming hormone therapy and the transgender community present a unique population and cannot be compared to hormone replacement therapy in postmenopausal females. Thus, it is crucial to consider the distinct and individual influences of hormones and hormone therapy in treatment.

## Clinical implications

While this review highlights an important body of evidence for the role of sex in cerebrovascular pathophysiology, injury diagnoses, treatment, and outcomes, changes in medical strategies at the point-of-care are critical to invoke meaningful improvements for patients. It is important that health professionals consider sex, and its potential implications, in their treatment. Females are at risk of receiving preventative medications and in-hospital resources less often than males, suggesting disparities in access to healthcare systems, differences in health-seeking behavior, and differing attitudes toward acute neurological illnesses.^3,9,122^

As this area of evidence-based medicine is relatively new, recommendations for changing practice are largely missing; thus, we cannot recommend specific treatments for males versus females until this research exists. Further research into the effects of sex and sex-specific hormones on cerebrovascular regulation may lead to important clinical translation. Regardless, increased awareness of sex differences in disease risk, response to treatments, and outcomes is the first step in the advancement of sex-specific evidence-based medicine, which will enable better, more personalized care for all. Consideration of sex in development of clinical trials, analysis design, and biomarker development is crucial. In the future, adoption of such evidence-based research is essential for developing flexible frameworks for intensive-care medicine and influencing clinical guidelines, public health regulations, and educational initiatives.

### Considerations

It is important to acknowledge this review is narrative in nature; thus, it is limited by using a non-systematic review of the literature. While we aimed to cross-reference major epidemiologic studies, systematic reviews, and key clinical trials, and present conflicting evidence where it exists, we recognize these limitations. The area would benefit from a systematic review in the future; however, the current review identifies many gaps in the current literature that should be addressed first to provide additional merit and evidence for conducting a formal systematic review.

## Conclusions

In this review, we summarize the impact of biological sex on cerebrovascular physiology and pathophysiology. Future study is required to better understand how hormones and variations in hormones with menopause, contraception, and hormone therapy all influence cerebrovascular physiology in both health and disease, which will likely lead to advances in treatment that may be sex specific. Understanding the pathophysiology that underpins these sex differences is paramount to developing new neuroprotective therapies that may be sex-specific and improve outcomes for both males and females. While difficult to completely isolate, this review focused primarily on the effect of sex as a biological variable. However, the role of gender and its intersection with sex are paramount in how an individual may experience disease and the ensuing treatments and outcomes and are vital considerations in both research and medical care.
